# Sporting activity is reduced following medial reefing performed for patellar dislocation: A retrospective case series of 144 patients with a minimum follow-up of 24 months

**DOI:** 10.1186/s12891-019-2400-z

**Published:** 2019-01-22

**Authors:** C. Ihle, J. Maurer, P. Ziegler, U. Stöckle, A. Ateschrang, M.-D. Ahrend, S. Schröter

**Affiliations:** 1 Department of Traumatology and Reconstructive Surgery, BG Trauma Center Tübingen, Eberhard Karls University Tübingen, Schnarrenbergerstr. 95, 72076 Tübingen, Germany; 2 AO Research Institute Davos, Clavadelerstr. 8, 7270 Davos, Switzerland

**Keywords:** Medial reefing, Sporting activity, Return to sports, Patellar dislocation

## Abstract

**Background:**

Patellar dislocation is common in young and active patients. The purpose of this study was to determine sporting activity following the medial reefing of patellar dislocation.

**Methods:**

One hundred forty-four patients with objective patellar dislocation were treated between 2004 and 2013. Three groups were analyzed retrospectively with a minimum follow-up of 24 months: (1) primary dislocation that was treated with medial reefing without a recurrent dislocation until the day of follow-up (*n* = 74), (2) primary dislocation that was initially treated with medial reefing but with a recurrent dislocation until the day of follow-up (*n* = 44), and (3) medial reefing after failed conservative treatment (*n* = 26). Sporting activity was assessed using a widely-used sporting activity questionnaire and the Tegner score prior to the injury and at the follow-up (58.7 ± 22.6 months after the injury). Clinical outcomes were assessed using IKDC and Kujala score.

**Results:**

The Kujala score was 94.7 ± 9.3 for Group 1, 84.1 ± 16.6 for Group 2 and 93.4 ± 9.7 for Group 3. IKDC at the time of follow-up was 97.2 ± 9.3 for Group 1, 86.1 ± 14.6 for Group 2 and 95.1 ± 11.1 for Group 3. 91.9% of Group 1 and 92.3% of Group 3 were active in sports prior to their injuries and at the time of the follow-up. In Group 2, sporting activity reduced from 81.8 to 75.0%. In all groups, a shift from high performance to recreational sports was found.

**Conclusions:**

Despite good clinical results, sporting activity was reduced following patellar dislocation treated with medial reefing. Also, a shift from engagement in high- to low-impact sports among the participants was noted.

## Background

Determining sporting activity level following knee injuries among active patients is important and sport activity levels have been reported for people with different injuries [1–4]. Patellar dislocation is common among young and active patients [5–7]. These patients tend to try to return to their previous sports and want to know their chances of returning to high- or low-impact sporting activities. Operative reconstruction of the medial patellofemoral ligament (MPFL) is widespread following patellar dislocation, and there are many publications about the outcomes of this treatment option [8–10]. In contrast, medial reefing is less evaluated and there is limited data about the patient-specific sporting activity following this procedure [11, 12].

The aim of this retrospective study was to analyze returns to sporting activity after patellar dislocation and treatment with medial reefing. The collected sample size was divided into three subgroups: (1) patients who received operative treatment with medial reefing following primary patellar dislocation and who were not affected by a recurrent dislocation until the time of the survey, (2) patients who were treated with medial reefing and suffered from recurrent dislocation until the time of the survey, and (3) patients who were initially treated conservatively but suffered from a recurrent patellar dislocation. The recurrent patellar dislocation was treated with medial reefing. It was hypothesized that sporting activity is reduced following primary and recurrent patellar dislocation which were treated with medial reefing (1) and that most of the patients would shift to engaging in low-impact sports from high-impact sports (2), especially following recurrent patellar dislocation.

## Methods

### Study population and data acquisition

**Fig. 1:**
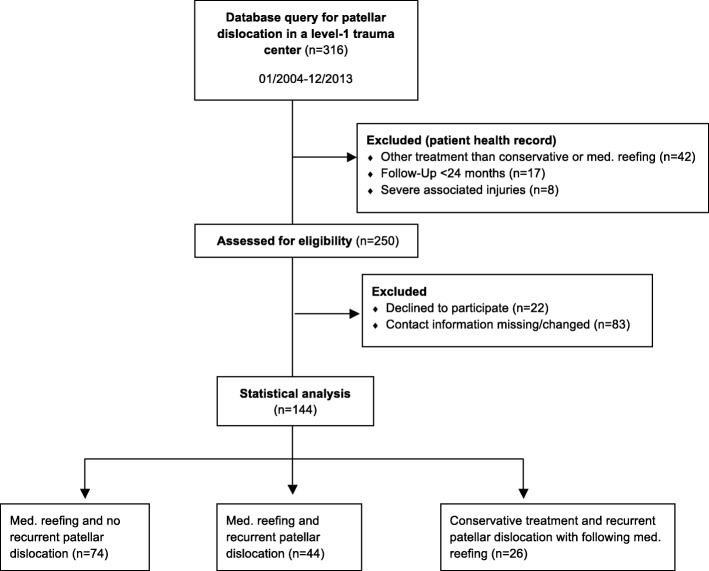
Patient flow-chart according to STROBE Standards

The study protocol (195/2014BO2) was approved by the local ethics committee. Informed consent was obtained from all included patients. Between 2004 and 2013, 316 patients were documented with primary patellar dislocation in a Level 1 trauma center in Western Europe. Patients with a follow-up time of less than 24 months were excluded. Surgical treatment of a patellar dislocation was recommended to patients with a lesion of the MPFL diagnosed with an MRI (magnetic resonance imaging) or to patients with a recurrent dislocation after conservative treatment. Recurrent dislocation was defined as a second or subsequent dislocation. All patients that were treated conservatively or using MPFL reconstruction or an osteotomy were excluded from the study. A patient flow chart is presented in Fig. 1 according to STROBE (strengthening the reporting of observational studies in epidemiology) standards. All patients that received operative treatment by medial reefing (*n* = 144) were finally grouped into three study arms. Each group (Table 1) was analyzed for sporting activity, working activity and clinical outcome.

- Group 1: Patients who received operative treatment (medial reefing) of a primary patellar dislocation and experienced no further recurrent patellar dislocation until the day of follow-up (*n* = 74)
- Group 2: Patients who received operative treatment (medial reefing) of a primary patellar dislocation and experienced a further recurrent patellar dislocation until the day of follow-up (*n* = 44)
- Group 3: Patients who received conservative treatment of a primary patellar dislocation and experienced recurrent patellar dislocation and subsequent operative treatment (medial reefing) (*n* = 26)

**Table 1:** Patient characteristics of all three groups

	Follow-up after first treatment	Gender	Age at primary dislocation	Age at recurrent dislocation	Time from dislocation to operative treatment	Affected side	Cause of last dislocation
Group 1 *n* = 74	59.3 ± 22.4 months	64.9% men 34.1% women	20.1 ± 7.7 years		26.4 ± 23.2 days	55.4% left 44.6% right	62.2% sport accident 27.0% activities of daily living 8.1% working accidents 2.7% traffic accidents
Group 2 *n* = 44	57.7 ± 24.5 months	54.5% men 45.5% women	17.5 ± 7.9 years	22.3 ± 9.1 years	58.4 ± 70.3 days	47.7% left 52.3% right	52.3% activities of daily living 38.6% sport accidents 6.8% working accidents 2.3% unknown reason
Group 3 *n* = 26	58.7 ± 20.3 months	35.0% men 65.0% women	16.6 ± 7.3 years	20.5 ± 8.5 years	61.5 ± 66.1 days	85.7% left 14.3% right	61.5% sport accidents 38.5% activities of daily living

### Sporting activity and clinical outcome

Sporting activity level was assessed using a commonly-used questionnaire, as well as the Tegner activity scale. The modified sporting activity questionnaire was previously used several times to determine changes in 20 predefined sporting and recreational activities [1, 2, 13, 14]. The questionnaire was assessed at the time of injury and at the time of the follow-up survey. Activity level was classified into the following groups: professional sports, competitive sports, recreational sports, and no sports. If the patient was sport-active, then the kind of sport as well as the weekly sporting frequency and duration, the number of different sports engaged in, and the length of each activity were documented. Additionally, all activities were classified into two groups: high-impact activities (soccer, handball, basketball, volleyball, squash, badminton, tennis, skiing, running, inline skating, dancing) and low-impact activities (cycling, hiking, Nordic walking, aerobics, golf, swimming, gymnastic, horse riding, fitness training). Furthermore, the patient was asked for information about injury-related changes regarding specific sporting activities. The REFA classification was used to evaluate working activity and intensity prior to the injury and at the time of the follow-up observations [15]. The REFA classification contains of five grades from 0 to 4: (0) without, (1) small, (2) moderate, (3) hard, and (4) most heavily physical strain. Kujala scores and IKDC questionnaire results were used to evaluate clinical outcomes. These scores were assessed at the follow-up appointment.

### Surgical technique and postoperative rehabilitation

**Fig. 2:**
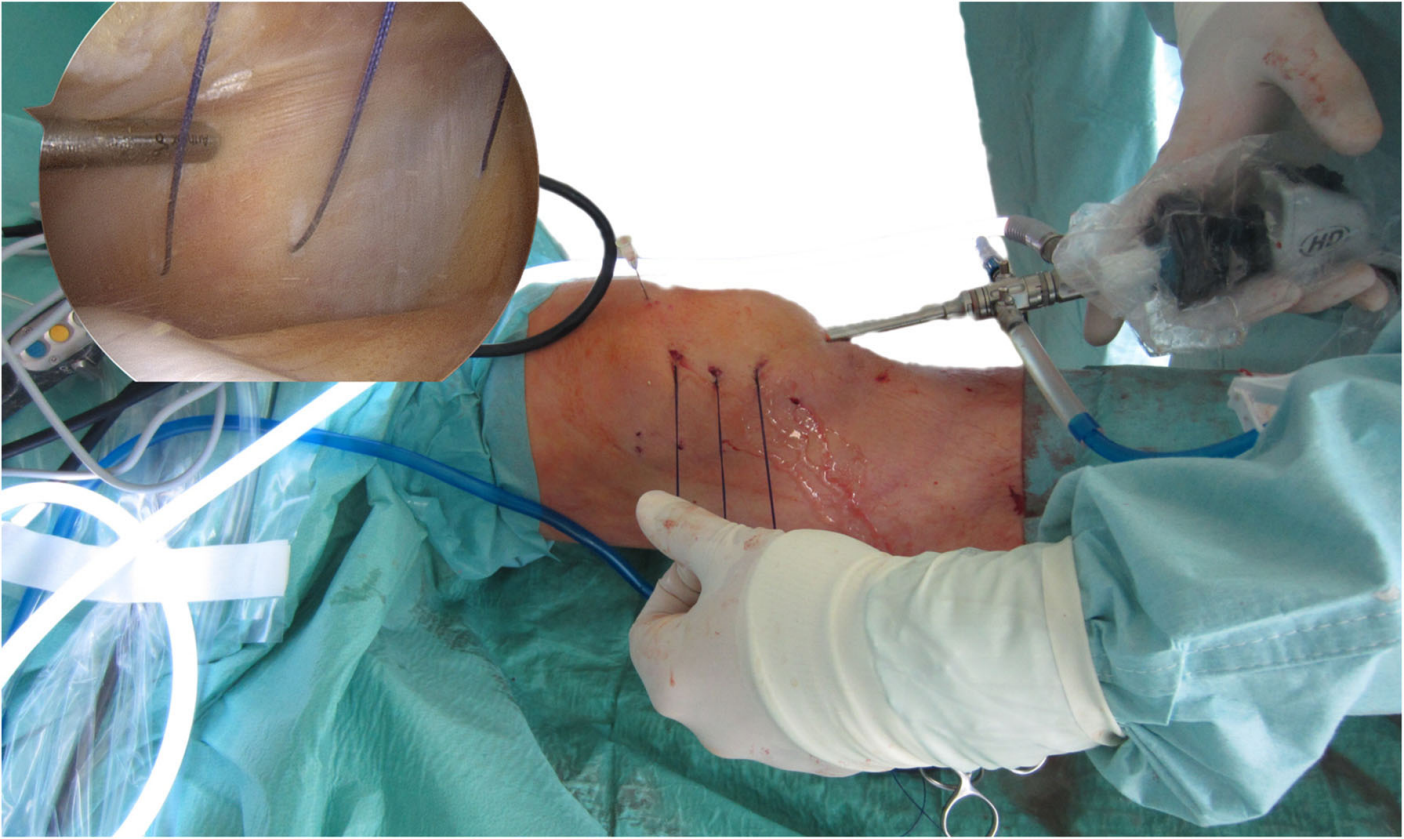
Arthroscopic medial reefing. The sutures are already percutaneously passed extra-articular. The arthroscopic view shows the position of the sutures as well as the location of shaving

Arthroscopic medial reefing was performed under general or spinal anaesthesia. The patient was positioned in supine position. The tourniquet was inflated. A standard anterolateral arthroscopic portal was established. This was followed by the establishment of an anteromedial portal. After the diagnostic arthroscopy, the medial capsule was gently shaved to improve the intraarticular healing process. A Vicryl CTX sized 0 suture (Johnson & Johnson Medical GmbH, Ethicon Germany, Norderstedt, Germany) was placed at the proximal, medial border of the patellar directly next to the bone and taken out approximately 3 cm posterior-medial (Fig. 2). A small skin incision at the insertion of the suture was performed, and the suture was passed percutaneously into the layer between the capsule/medial retinaculum and the subcutaneous layer [16]. Then, a second and a third suture were positioned 1.5 cm distally to the above suture using the same technique. The edges of each suture were tied and the knots were tightened at 30° of knee flexion after the removal of the intraarticular fluid. The wounds were closed using skin sutures. This was followed by sterile wound dressing and elastic compressive casting.

Full weight-bearing was allowed immediately after the surgery. After the first week a knee bandage for active muscular and passive patellar medialization was recommended. The active and passive range of motion were limited between 0° and 60° for the first three weeks, and 0° and 90° for the following three weeks. After these 6 weeks, full range of motion was permitted and intense physiotherapy, including coordinative training and strength training, was performed. The patients were allowed to return to engagement in sports after 12 weeks.

### Statistics

Time between specific events, evaluated scales as well as evaluated scores were presented as mean ± standard deviation (minimum-maximum). Normal distribution was tested using Kolmogorov–Smirnov test. In case of significant deviations from normal distribution, non-parametric methods were used for further testing. Kruskal–Wallis test was used to compare more than two independent samples. The Mann–Whitney U test was applied to quantitatively compare two independent groups. Ordinally- as well as nominally-scaled values were presented as percentages and absolute frequencies, whereas two of each of these values were compared in contingency tables. Association was tested with the chi-square test. The significance level was 5% and statistical testing was carried out with IBM SPSS Statistics 23 (SPSS Inc., IBM Company, Chicago, IL, USA).

## Results

### Patient characteristics of the three subgroups

The patient characteristics of all three groups are summarized in Table 1. In group 1, the primary dislocation was a lateral dislocation and repositioning was carried out without professional help in 62.2% of the cases. In 55.4% (*n* = 41) of the cases, isolated medial reefing was done. In 18.9% of the cases (*n* = 14) additional flake resection was needed. In 13.5% of the cases (*n* = 10), additional flake refixation was carried out, in 5.4% of the cases (n = 4) additional micro-fracture treatment was carried out, in 2.7% of the cases (*n* = 2) additional autologous chondrocyte transplantation (ACT) was performed and in 4.1% of the cases (*n* = 3) other additional procedures were carried out.

In group 2, the patients suffered from recurrent dislocation 31.8 ± 52.7 months after the primary treatment. All suffered from a lateral primary dislocation that repositioned spontaneously in most cases (81.8%). For recurrent dislocation, 25% (*n* = 11) of the patients were treated conservatively. For 22.7% (*n* = 10) of the patients, isolated reconstruction of the medial patellofemoral ligament was done, for 4.5% (*n* = 2) combined with ACT, for 2.3% (*n* = 1) combined with flake resection and in 2.3% (*n* = 1) combined with micro fracture treatment. Isolated deepening trochleaplasty was carried out in 13.6% of the cases (*n* = 6) and isolated medial reefing was carried out in 11.4% (*n* = 5) of the cases. A total of 11.4% (*n* = 5) received medial reefing combined with micro-fracture treatment and 2.3% (*n* = 1) received medial reefing combined with ACT. Isolated flake resection was done in 4.5% (*n* = 2) of the cases.

In group 3, the patients suffered from a recurrent patellar dislocation 13.0 ± 20.1 months after primary dislocation. The recurrent patellar dislocation was treated with medial reefing. All recurrent dislocations were lateral dislocations, and in 80.8% (*n* = 21) of the cases, no professional repositioning was necessary. All patients received medial reefing after a recurrent dislocation. Medial reefing and additional flake resection was performed after recurrent dislocation in six cases (23.1%), and in four cases (15.4%) it was performed in combination with flake refixation.

### Functional and clinical assessment

The mean Kujala score was 94.7 ± 9.3 (42–100) in Group 1, 84.1 ± 16.6 (39–100) in Group 2, and 93.4 ± 9.7 (68–100) in Group 3. Mean IKDC value at the time of follow-up was 97.2 ± 9.3 (50–100) in Group 1, 86.1 ± 14.6 in Group 2 (54–100) and 95.1 ± 11.1 (68–100) in Group 3. There was a statistically significant difference between Group 1 and 2 for the IKDC (*p* < 0.001; CI 95% [5.77;16.36]) and the Kujala score (*p* < 0.001; CI 95% [5.01; 16.13]), as well as between Group 2 and 3 for the IKDC (*p* = 0.006; CI 95% [− 15.86; − 2.10]) and the Kujala score (*p* = 0.007; CI 95% [− 16.51; − 2.06]). There was no difference between Group 1 and 3 for the IKDC (*p* = 1.000; CI 95% [− 8.43; 4.25]) and the Kujala score (*p* = 1.000; CI 95% [− 5.38; 7.94]).

The physical load levels at the workplaces of participants in all groups are described in Table 2. A total of 63.5% (*n* = 47) of patients in Group 1, 52.3% (*n* = 23) in Group 2 and 69.2% (*n* = 18) in Group 3 were students or not in a regular employment. No injury-related changes of the workplace were reported in Group 1 and 3. Regarding Group 2, one patient (2.3%) had to change jobs for injury-related reasons.

**Table 2:** Working activity according to the REFA classification

REFA	REFA pre-injury			REFA at follow-up		
	Group 1	Group 2	Group 3	Group 1	Group 2	Group 3
Grade 0	63.5% (47)	52.3% (23)	69.2% (18)	54.1% (40)	40.9% (18)	57.7% (15)
Grade 1	23.0% (17)	36.4% (16)	30.8% (8)	28.4% (21)	45.5% (20)	42.3% (11)
Grade 2	6.8% (5)	9.1% (4)	0.0% (0)	10.8% (8)	2.3 (1)	0% (0)
Grade 3	5.4% (4)	0.0% (0)	0.0% (0)	5.4% (4)	6.8% (3)	0% (0)
Grade 4	1.4% (1)	2.3% (1)	0.0% (0)	1.4% (1)	4.5% (2)	0% (0)

Grade 0: No employment

### Sporting activity

**Fig. 3:**
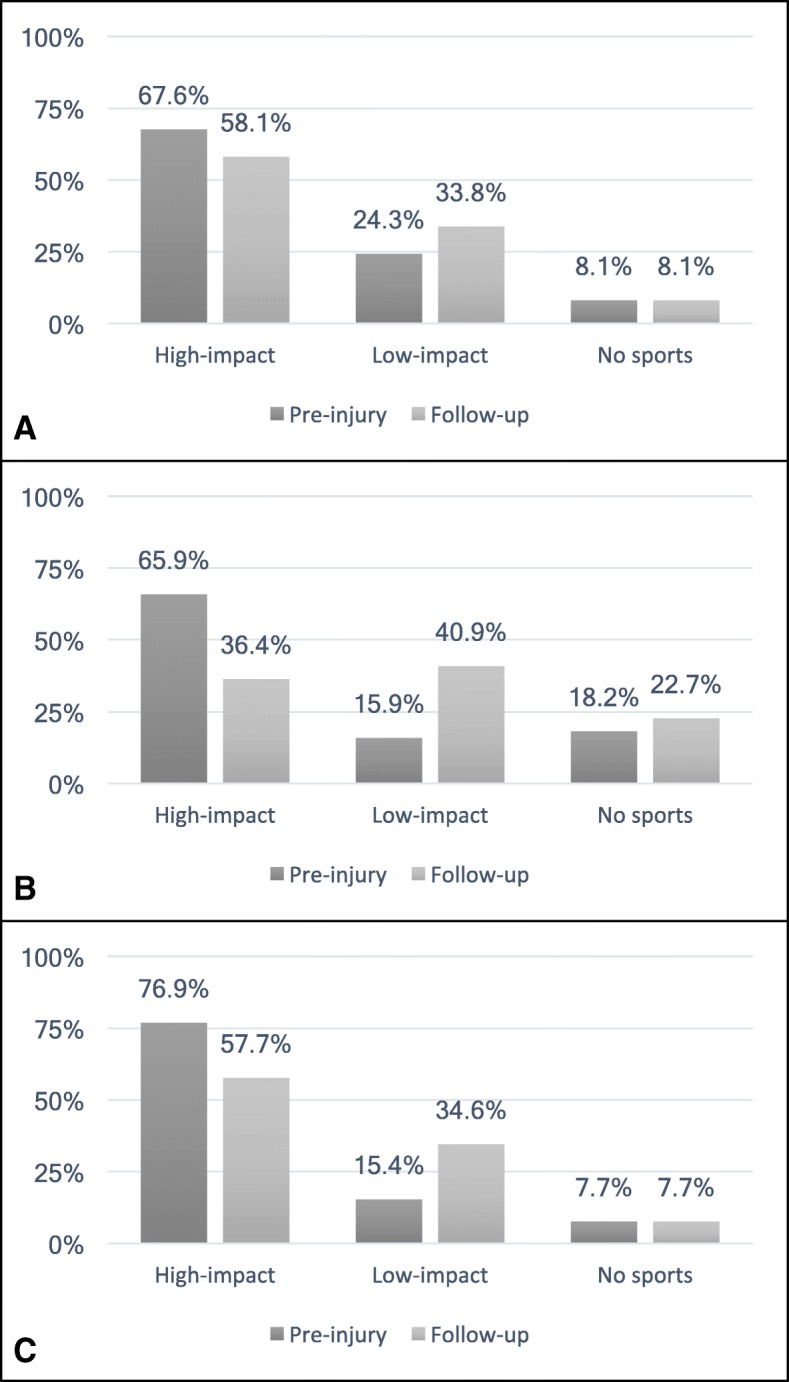
Changes in high- and low-impact sporting activities prior to the injury and at the time of follow-up. There is a shift from high- to low-impact sports in all groups (**a** Group 1, **b** Group 2, **c** Group 3)

**Fig. 4:**
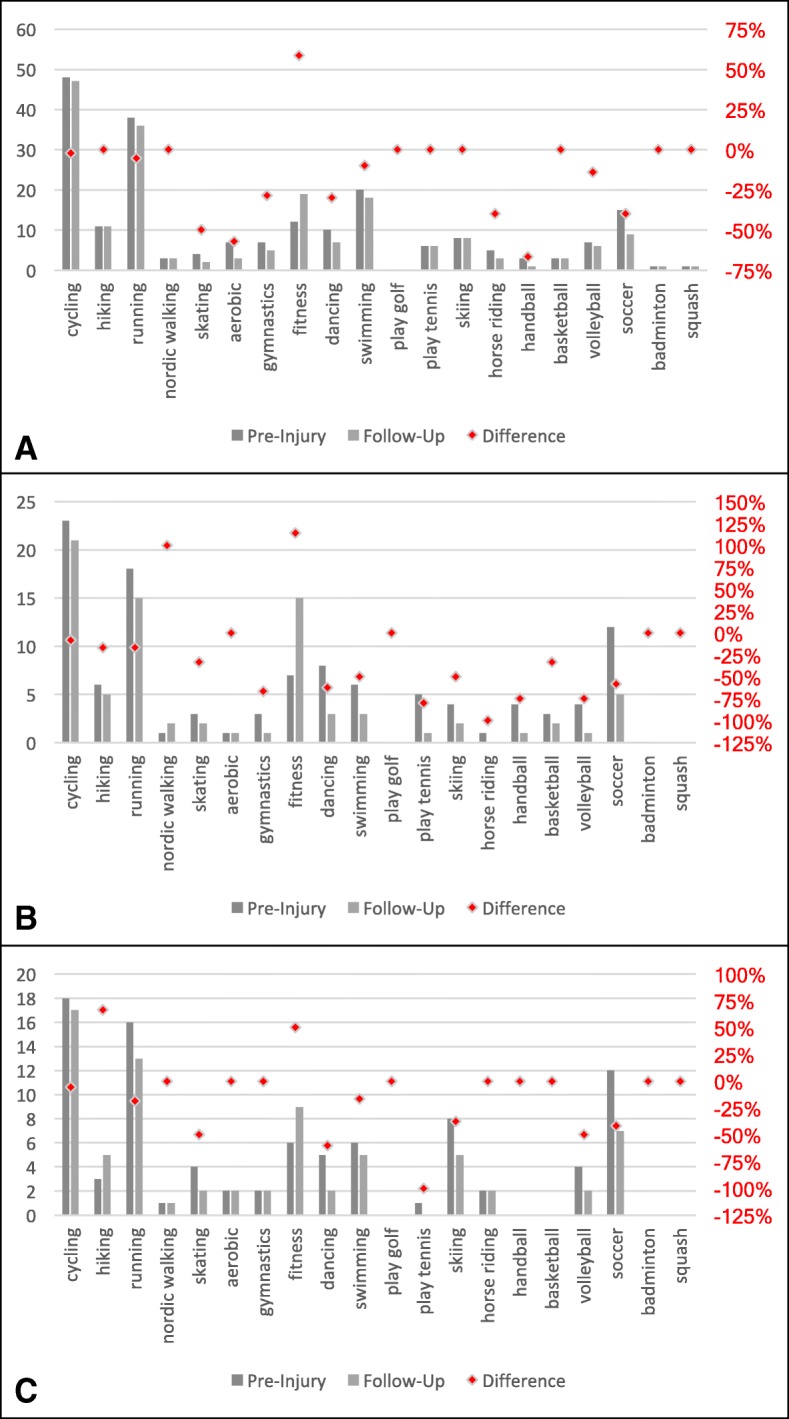
Sport-specific changes following primary patellar dislocation. Figure 4 describes the number of patients that attended the displayed sporting activities regularly prior to the injury and at the time of follow-up (**a** Group 1, **b** Group 2, **c** Group 3). Sport-specific changes (%) are displayed in red color

91.9% of the patients in Group 1 and 92.3% of the patients in Group 3 were active in sports prior to their injury as well as at the time of the follow-up survey. In Group 2 sporting activity reduced from 81.8 to 75.0%. Patients returned to sports after 22.2 ± 15.3 (3–52) weeks in Group 1, 22.7 ± 13.7 (6–52) weeks in Group 2 and 17.3 ± 13.1 (4–52) weeks in Group 3. There was a shift from high performance sports (professional and competitive sports) to recreational sports in all three groups. In each group, sporting frequency, the number of sports engaged in, and the durations of the activities were reduced at the time of follow-up (Table 3). There was also a shift from engagement in high-impact to low-impact sports in all groups, as described in Fig. 3. Discipline-specific changes are presented in Fig. 4. Injury-related changes regarding sports type were experienced by 17.6% of Group 1, 45.5% of Group 2, and 30.8% of Group 3.

**Table 3:** Sporting activity prior to the injury and at the time of follow-up survey

	Groups	Pre-injury	Follow-up	*p*-value
Sporting frequency	1	2.5 ± 1.5	2.1 ± 1.3	*p* < 0.001
	2	2.8 ± 1.4	2.1 ± 1.3	*p* = 0.003
	3	3.1 ± 1.5	2.4 ± 1.3	*p* = 0.005
Number of sports	1	2.4 ± 1.2	2.1 ± 1.1	*p* = 0.001
	2	2.8 ± 1.2	1.9 ± 1.1	*p* < 0.001
	3	3.3 ± 1.2	2.5 ± 1.2	*p* = 0.011
Duration of sporting activity	1	76.3 ± 23.9	69.2 ± 27.3	*p* = 0.001
	2	79.2 ± 26.5	64.1 ± 27.1	*p* = 0.001
	3	78.5 ± 19.9	68.3 ± 21.1	*p* = 0.085
Level of sporting activity				
Professional sports	1	1.4% (1)	0.0% (0)	
	2	2.3% (1)	2.3% (1)	
	3	7.7% (2)	0.0% (0)	
Club sports	1	43.2% (32)	29.7% (22)	
	2	47.7% (21)	15.9% (7)	
	3	26.9% (7)	19.2% (5)	
Recreational sports	1	47.3% (35)	62.2% (46)	
	2	31.8% (14)	56.8% (25)	
	3	57.7% (15)	73.1% (19)	
No sports	1	8.1% (6)	8.1% (6)	
	2	18.2% (8)	25.0% (11)	
	3	7.7% (2)	7.7% (2)

Differences in the pre-injury and the postoperative sporting activity levels of patients with and without concomitant injuries, and differing follow-up times, were analyzed in each group. No significant differences between patients with and without concomitant injuries regarding sport duration, the number of sports engaged in and frequency of sporting activity were found between the three groups. Moreover, patients with a follow-up of 2 to 5 years were compared to patients with a follow-up of 5 to 10 years: No significant differences in sport duration, the number of sports engaged in, and the frequency of sporting activity were found in each of the three groups.

The Tegner activity score was also used to assess changes in the sporting activity. The Tegner score decreased in all three groups. The mean pre-injury score was 5.7 ± 1.9 (2–10) in Group 1, 5.8 ± 2.3 (0–10) in Group 2, and 6.3 ± 2.1 (0–10) in Group 3. The mean postoperative score were 5.1 ± 1.7 (2–9) in Group 1, 4.4 ± 1.9 (0–10) in Group 2, 4.9 ± 1.9 (0–8) in Group 3. Totals of 75.7% of Group 1, 54.5% of Group 2, and 57.7% of Group 3 returned to their pre-injury activity levels. There were statistically-significant differences in the Tegner activity score in Group 1 (*p* < 0.001), 2 (*p* < 0.001) and 3 (*p* = 0.003).

## Discussion

The most important finding supports the hypothesis that sporting activity is reduced after patellar dislocation and treatment with medial reefing. There was a significant shift from engagement in high-performance and high-impact sports to recreational and low-impact sports. In the present study, all patients with operative treatment without recurrent dislocation and all patients with medial reefing after conservative treatment returned to sporting activity within six months after operation. Notably lower return-to-sport rates were observed in patients who suffered from a recurrent dislocation following medial reefing. Injury-related changes in disciplines were reported by 17.6% (Group 1) to 45.5% (Group 2) of patients. Considering that most patients are young and that an athlete’s motivation to undergo a surgery is often to return to playing sports at the same level that they once did [17], the return-to-sport rate is low. Other factors, such as changes in life-style or occupational demands, can influence whether an individual continues to participate in sports after surgery. However, no significant differences were found between the frequency and duration of the sport activity and the number of sports engaged in between patients with follow-up times of less and more than 5 years.

The previously-available literature regarding sporting activity after medial reefing for the initial treatment of patellar dislocation, as well as sporting activity after medial reefing in failed initial conservative treatment, is limited. However, the literature provides detailed results regarding returns to sports after reconstruction and repair of the medial patellofemoral ligament in recurrent patellar dislocation [18–20]. A recent meta-analysis by Schneider et al. [18] described superior results compared to the present study with a pooled rate of returns to the pre-injury sport level in 84.1% of patients who received isolated MPFL reconstruction [18]. A review by Matic et al. [21] about the return-to-sport rates in a heterogeneous sample with combined MPFL reconstruction, MPFL repair, and patients with medial retinaculur repair/plication reported a return-to-sport rate to the previous sport level of 90%. Ambrozic et al. described returns to sport in 88.5% of patients following MPFL reconstruction; 69.6% of patients returned to the same level [22]. Also, Lippacher et al. [23] reported similar return-to-sport rates after isolated MPFL reconstruction. All patients who participated preoperatively in sports returned to sports after a minimum follow-up of two years. An equal or higher sport level was reached in 53% of patients. Nelitz et al. [24] found that 60% of patients returned to their previous sport activities and 60.7% reached the same or even a higher levels than preoperatively after combined trochleoplasty and MPFL reconstruction. In contrast, only 26.4% of the conservatively-treated patients without further dislocations reported that they were able to return to their desired sport activities without limitations following their dislocation [25].

In the present study, the clinical outcomes were sufficient for patients who were not suffering from recurrent dislocation. This highlights the fact that patients can be successfully treated with medial reefing. Despite these positive results, 37.3% of the patients experienced a recurrent dislocation after initial medial reefing. These patients had inferior clinical outcomes (84.1 ± 16.6 points) compared to those in Group 1 and 3. A recent analysis of patient outcomes after medial reefing in combination with lateral release with a median follow-up time of 9.7 years revealed poor results with high failure rates of 42% after 5 years and 52% after 10 years [11]. The Kujala knee scores (88.2 ± 13.5) in 23 knees treated with mini-open medial reefing and arthroscopic lateral release reported by Nam et al. [26] were similar to the findings of the present study. However, Nam et al. [26] reported that only 8% of patients suffered from recurrent dislocation or subluxation [26]. Miller et al. [27] also found lower re-dislocation or subluxation rates than those found in the present study. In 25 knees, no recurrent event occurred.

Schneider et al. [18] reported that patients who were treated with isolated MPFL had a pooled Kujala score of 85.8 points, which is similar to the results of the present study. However, other studies reported inferior results for medial reefing compared to MPFL reconstruction. Zhao et al. [28] compared the clinical outcomes of adult patients who underwent medial retinaculum plication and MPFL reconstruction for recurrent patellar instability. After a follow-up period of 5 years, significantly better results were found for the MPFL reconstruction than for the medial retinaculum plication (Kujala score: 87.4 ± 5.7 vs. 73.8 ± 5.5, respectively). Based on the present study, no conclusion can be drawn about which technique is preferable and whether better outcomes can be achieved through early surgical intervention after the dislocation. However, the aim of the present study was to analyze the returns to sports after medial reefing and fill a gap in the previous literature.

The relatively low clinical outcomes and the lower return-to-sports rate in Group 2 of the present study emphasize the need to reduce the risk of recurrent dislocations, which occurred 12 months after the initial surgery, on average. To prevent patients from experiencing such dislocations, the correct treatment selection is needed to address the individual anatomy of each patients. This decision is difficult and controversial [29, 30]. In this study, the surgery for treating the recurrent dislocation was individually selected and varied highly between the cases to address the anatomical risk factors of patellar re-dislocations. This led to various secondary treatments being selected for patients in Group 2. This resulted in more invasive secondary treatments, but no recurrent dislocations occurred in the following. This underlines the necessity of individualized and detailed preoperative planning to improve patient outcomes. In patients who experienced a recurrent dislocation, a long-leg weight-bearing x-ray, patella defilée x-rays, a torsional CT and an MRI should be performed to assess the anatomical risk factors that are associated with patellar dislocations [31].

Besides the correct treatment selection, also the return-to-sport decision-making process needs to be comprehensively evaluated to prevent recurrent dislocations. The stability of the lower limb and equally distributed muscle strength are especially important to avoid re-dislocation. Stop-and-go-movements, sudden changes in direction, and running on uneven ground are activities which increase the risk of re-dislocation [17]. As suggested by Ménétrey et al. [17] the decision of when to return to sports should be based on six criteria: The patient should have no pain, no effusion, no patellofemoral instability, full range of motion, symmetrical strength, and excellent dynamic stability. By considering these criteria and by enhancing the postoperative rehabilitation process, higher return-to-sports rate and lower recurrent dislocation rates following medial reefing may be achieved.

There are several limitations of the study, that have to be discussed. The sample is heterogeneous, especially concerning of concomitant cartilage lesions, which might have influenced the return-to-sport activities of these patients. However, no significant differences were found between patients with and without concomitant injuries in each group. The study is limited by the response rate of 59%, which weakens the validity and reliability of the study. Selection bias is possible if study participants had different return-to-sport outcomes than patients who were not eligible or declined to participate in the survey. However, the response rate is above the recommended 50% minimum to minimize bias effects [32], and it is comparable to those of other surveys on returns to sports after knee injuries [33]. Recall bias could also influence the results due to the self-reporting of the primary outcome measurement (return to sport) and the retrospective study design that relies on patients to accurately self-report these measurements form upwards 5 to 10 years earlier. Due to our hospital’s focus on surgical treatment, only patients with recurrent dislocations following primary conservative treatment returned to our clinic. We cannot describe of what the conservative treatment consisted of in detail. Hence, returns to sport after conservative treatments could not be analyzed.

Besides these limitations, the strengths of this study are its large cohort and the detailed analysis of return-to sport outcomes after patella reefing, which was not been previously reported on. Young and sport-active patients who suffer from patellar dislocation try to return to their previous sports and want to know their chances of doing so. Based on the presented results, estimations of the return to sports following initial operative treatment (medial reefing) with or without further dislocation and regarding patients with initial conservative treatment and recurrent dislocation treated by medial reefing can be made. Considering the high rate of recurrent dislocations and the poor clinical and functional outcomes of patients who experience recurrent dislocations after medial reefing, recurrent dislocations after surgical treatment should be strictly avoided by initially analyzing and treating all pathological aspects of the malalignment.

## Conclusions

Despite good clinical results, sporting activity level decreased in all patients after medial reefing. A shift from professional and competitive to recreational sports as well as from high- to low-impact sports was noted.

## Abbreviations

ACT: Autologous chondrocyte transplantation
CI: Confidence interval
IKDC: International knee documentation committee
MPFL: Medial patellofemoral ligament
MRI: Magnetic resonance imaging
REFA: “Verband für Arbeitsgestaltung, Betriebsorganisation und Unternehmensentwicklung e. V.”
SD: Standard deviation
STROBE: Strengthening the reporting of observational studies in epidemiology
